# Overcoming language barriers in pediatric care: a multilingual, AI-driven curriculum for global healthcare education

**DOI:** 10.3389/fpubh.2024.1337395

**Published:** 2024-02-22

**Authors:** Fouzi Benboujja, Elizabeth Hartnick, Evelyn Zablah, Cheryl Hersh, Kevin Callans, Perla Villamor, Phoebe H. Yager, Christopher Hartnick

**Affiliations:** ^1^Department of Otolaryngology, Mass Eye and Ear, Harvard Medical School, Boston, MA, United States; ^2^Brown University, Providence, RI, United States; ^3^Pediatric Airway, Voice and Swallowing Center, Massachusetts General Hospital for Children, Boston, MA, United States; ^4^Massachusetts General Hospital for Children, Boston, MA, United States; ^5^Hospital Serena del Mar, Cartagena, Colombia; ^6^Hospital Infantil Napoleón Franco Pareja, Cartagena, Colombia; ^7^Pediatric Intensive Care Unit, Massachusetts General Hospital, Harvard Medical School, Boston, MA, United States

**Keywords:** pediatric, global health, generative language models, digital health, education, artificial intelligence (AI), curriculum, healthcare

## Abstract

**Background:**

Online medical education often faces challenges related to communication and comprehension barriers, particularly when the instructional language differs from the healthcare providers' and caregivers' native languages. Our study addresses these challenges within pediatric healthcare by employing generative language models to produce a linguistically tailored, multilingual curriculum that covers the topics of team training, surgical procedures, perioperative care, patient journeys, and educational resources for healthcare providers and caregivers.

**Methods:**

An interdisciplinary group formulated a video curriculum in English, addressing the nuanced challenges of pediatric healthcare. Subsequently, it was translated into Spanish, primarily emphasizing Latin American demographics, utilizing OpenAI's GPT-4. Videos were enriched with synthetic voice profiles of native speakers to uphold the consistency of the narrative.

**Results:**

We created a collection of 45 multilingual video modules, each ranging from 3 to 8 min in length and covering essential topics such as teamwork, how to improve interpersonal communication, “How I Do It” surgical procedures, as well as focused topics in anesthesia, intensive care unit care, ward nursing, and transitions from hospital to home. Through AI-driven translation, this comprehensive collection ensures global accessibility and offers healthcare professionals and caregivers a linguistically inclusive resource for elevating standards of pediatric care worldwide.

**Conclusion:**

This development of multilingual educational content marks a progressive step toward global standardization of pediatric care. By utilizing advanced language models for translation, we ensure that the curriculum is inclusive and accessible. This initiative aligns well with the World Health Organization's Digital Health Guidelines, advocating for digitally enabled healthcare education.

## 1 Introduction

In pediatric healthcare, each stage of the patient journey—from initial assessments to post-treatment convalescence to eventual safe discharge home—poses its own set of unique challenges. These complexities are universal, transcending geographical and cultural boundaries (1). The COVID-19 pandemic has further spotlighted the crucial impact of cultural and linguistic differences on various aspects of healthcare (2). From determining patient access (3) to affecting continuity of care (4), these elements have been empirically shown to significantly influence health outcomes (5–10), especially during critical health events such as global pandemics (11–13).

Building on the understanding of the role of cultural and linguistic elements, a particularly pressing challenge arises in the domain of healthcare education (1, 14). Caregivers, parents, and healthcare professionals often face communication and comprehension barriers when the predominant language of instruction is not their native language (15). This issue becomes especially acute in pediatric care, where accuracy and in-depth understanding are imperative.

Considering the well-documented gaps in communication within healthcare settings (15, 16) and their subsequent ramifications (4, 17), there is a clear and pressing need for multimedia resources that are not just comprehensive but also culturally and linguistically tailored. In addressing this need, CareWays Collaborative was founded in 2022 with a mission to partner with health teams around the globe to transform the culture and delivery of surgical care so that all patients can achieve the most favorable outcome. This group of healthcare professionals based in Boston and affiliated with Massachusetts Eye and Ear (MEE) and Massachusetts General Hospital (MGH), aims to create and disseminate educational materials that bridge communication gaps and distribute knowledge effectively across diverse healthcare settings. This effort aligns with the World Health Organization's Digital Health Guidelines, which underscores the importance of leveraging digital interventions for health worker training and education (18). In this vein, CareWays Collaborative created video-based educational content aided with artificial intelligence (AI) to ensure both clarity and cultural relevance for various healthcare provider and caregiver demographics.

AI is playing an increasing role in bridging gaps in healthcare education (19, 20). With the emergence of diverse techniques, from computer vision (21, 22) and data analytics (23) to Natural Language Processing (NLP) (24, 25), AI offers promising solutions to longstanding challenges related to language and cultural barriers. Notably, innovative tools such as Nvidia's Riva (26) and state-of-the-art generative language models—including OpenAI's ChatGPT 4 (27), Meta's LLaMA 2 (28), and Microsoft's PaLM 2 (29)—are enabling real-time translation. This advancement not only makes medical education more accessible but also emphasizes the essential role of AI in enhancing these resources.

Drawing on a decade of empirical research, a curriculum has been developed using artificial intelligence and voice cloning technologies to address communication gaps in pediatric healthcare. It offers insights into effective healthcare practices and highlights strategies to mitigate potential adverse events. In the form of an educational video series, the curriculum extends beyond recorded lectures or textbook material by including live demonstrations of important techniques and procedures used in operating rooms and at patients' bedsides. The videos focus on very subspecialized critical care and surgical techniques geared toward critical care nurses, physicians and respiratory therapists, pediatric anesthesiologists and surgeons, and pediatric speech-language pathologists—all crucial members of the specialized team caring for pediatric patients with airway pathology and/or critical illness. Each of these videos has been organized into sub-specialty-specific chapters within the Canvas educational platform to serve as a resource. For example, there are 19 instructional videos for Pediatric Intensivists, including video instruction on “how to tape and secure an endotracheal tube,” “how to dress, access and care for a central venous catheter,” and “how to prevent ventilator-related pneumonias.” These chapters were developed after qualitative interviews with intensivists in low/middle-resource countries (30). By dividing the content into these targeted volumes, we believe that healthcare providers can access the in-depth material they need, tailored to their specific specialty, to address any knowledge gaps they wish to fill. The curriculum complements what you can read in a book and offers greater accessibility than a standard textbook—users can scan a QR code and watch a video for immediate guidance before diagnosing and assessing a patient, after an initial diagnosis when specific questions arise due to a knowledge or educational gap, or before performing a specific procedure.

This study undertakes an exploratory effort to leverage Generative Language Models (GLMs) for translation and voice synthesis to create a more inclusive and accessible educational environment in pediatric healthcare. The integration of software tools with AI functionalities forms a strategic blueprint for creating medical content with various languages and cultural backgrounds. While we initially targeted Spanish due to its global prevalence, the same methodology can be applied to diverse languages. This study primarily aims to provide an in-depth, sub-specialty-specific educational curriculum for critical care nurses, physicians, respiratory therapists, pediatric anesthesiologists, surgeons, and pediatric speech-language pathologists. It is designed to bridge educational gaps identified by their peers and to help improve the quality and care they can provide to their patients.

## 2 Materials and methods

Guided by the results of a learning behavior assessment survey administered to providers in a Central America-based public children's hospital within a low-resource setting (30), a narrated online video format was selected to ensure learning continuity even after the departure of a mission team. During two recent missions, first to Guatemala and then to Colombia, a comprehensive needs assessment was undertaken by an interdisciplinary team of experienced nurses (15), respiratory therapists (4), speech-language pathologists (2), anesthesiologists (10), intensivists (10), general practitioners (2), and surgeons (8) to identify gaps and process maps that could be addressed through video content (30). Using these insights, concise narrated videos were crafted at Mass General Brigham sites. These videos encapsulate key aspects of pediatric care, such as surgical procedures, perioperative care, patient journeys, and educational resources with best-practice guidelines for patients and caregivers. With durations ranging from 3 to 8 min, these high-quality videos, embedded with QR codes, were made freely available on learning platforms such as Canvas.

To improve accessibility, English narratives embedded in the videos were transcribed utilizing the “Dictate” function of Microsoft Word 365, prioritizing linguistic accuracy and contextual fidelity. Subsequently, OpenAI's Generative Pre-trained Transformer (GPT-4) was employed to facilitate the translation of content into Spanish. Native Spanish-speaking medical professionals, who were selected from the missions and well-versed in the challenges of low-resource settings, meticulously reviewed the translated content to ensure that the tone and sentiment aligned with the original material.

Building on the contextual translation, we focused on transmitting an authentic and relatable auditory experience. We collected voice samples from five candidates, each reciting the “Rainbow Passage” for 2 min. The Rainbow Passage is a standard reading passage used in speech-language pathology and voice assessment. It contains many sounds and intonations found in the English language, which improves the quality of the AI-driven synthetic voices. These recordings, captured via a mobile phone in lossless quality, were processed using the ElevenLabs Python library (elevenLabs_multilingual_v1). The model is derived from an extensive dataset of audio recordings and audiobooks designed to generate synthetic voices that closely emulate human speech characteristics. Voice synthesis was calibrated with tunable parameters emphasizing clarity (60%) and stability (30%). These parameters can be adjusted to fine-tune the synthetic speech output. Once synthesized, the audio was rendered as an mp3 file with a 44.1 KHz sampling rate. This synthesized audio track was integrated into the original video using Apple's iMovie software (v.10.3.8). To achieve optimal synchronization between visual and auditory elements, the playback speed of the synthesized audio was occasionally adjusted using the original English audio track as a reference.

**Figure 1:**
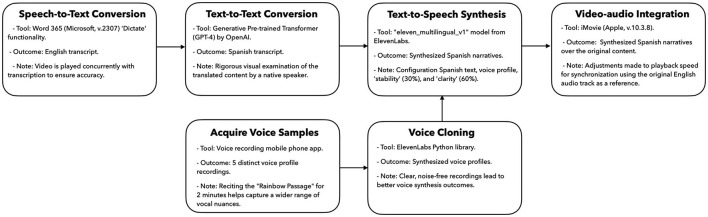
English to Spanish video translations using generative language models.

Figure 1 outlines the procedure for creating a 5-min educational video, which typically requires about an hour to produce. A significant part of this time is allocated to achieving narrative consistency. Our rationale for selecting the software tools used in this study was guided by their widespread accessibility, simplicity, and advanced capabilities. Additionally, many offer APIs that facilitate the adaptation of these tools to suit diverse workflows.

## 3 Results

**Figure 2:**
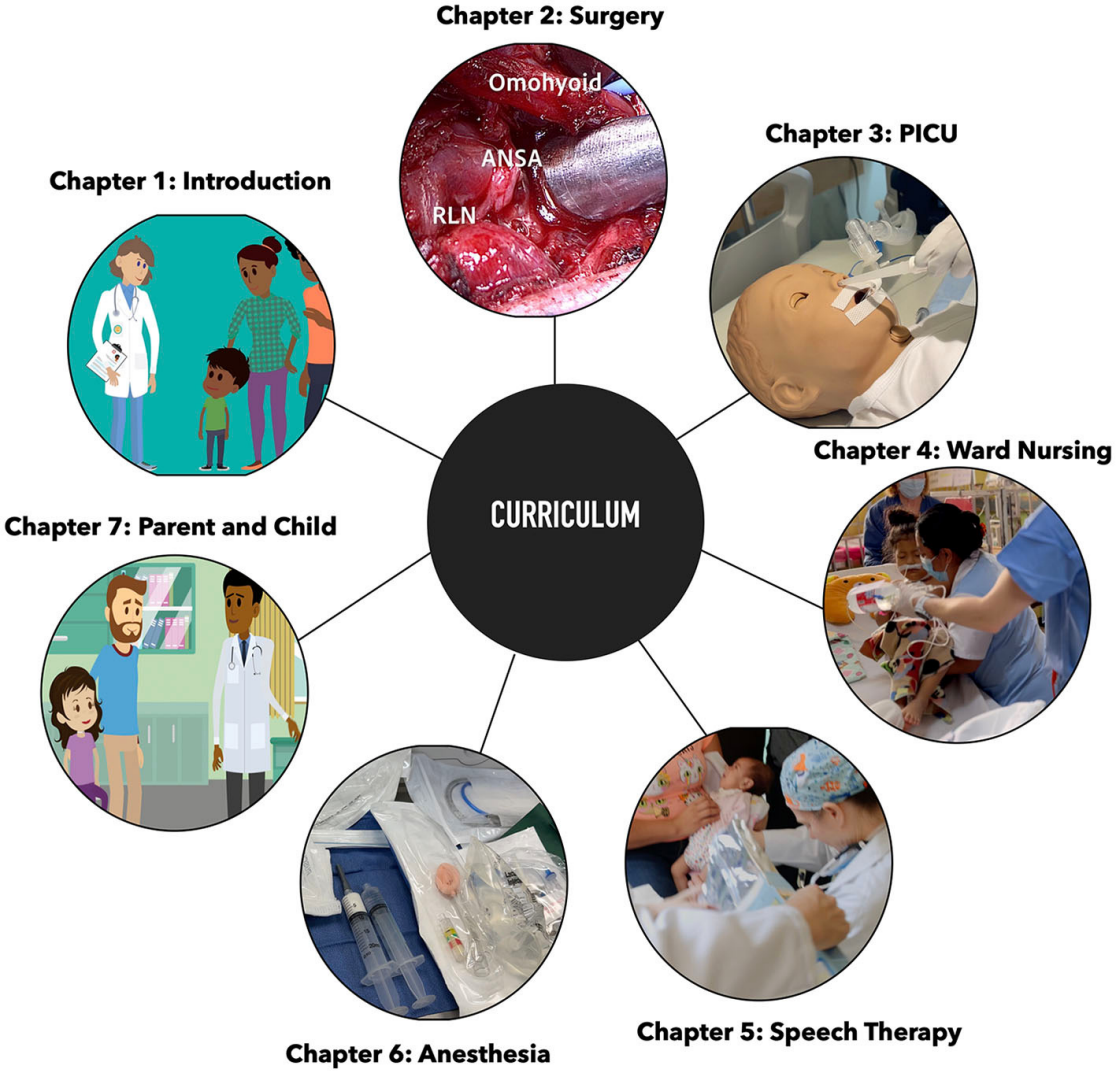
Snapshots from curriculum chapters.

We have developed a series of videos aimed at enhancing pediatric care, covering crucial areas from surgical interventions to nursing protocols. To enhance accessibility, these videos were translated into Spanish using state-of-the-art generative models, focusing on the primary demographic of North and Latin America, regions where our non-profit CareWays Collaborative frequently operates. Figure 2 displays snapshots from various curriculum chapters. For a detailed breakdown, refer to Supplementary Figure S1.

Subsequent sections outline the objectives and content of individual chapters.

*Introduction:* Provides an overview of the curriculum, highlighting the motivations and emphasizing the patient's journey across various stages of their care, including initial evaluation, surgery, intensive care, swallowing therapy, safe discharge planning, the transition from hospital to home, including caregiver responsibilities, and patient-caregiver interactions. The introduction is showcased in the English version (Supplementary Video S1) and in the machine-learning-enabled Spanish version (Supplementary Video S2). Although these examples are provided in English and Spanish, the methodology allows the creation of similar content with any other supported languages.

*Ward nursing:* Discusses transitional patient care from an intensive care unit to a general ward, emphasizing parent-centric care and individualized insights to improve patient outcomes. The nursing introduction here is presented both in the English version (Supplementary Video S3) and the machine-learning-enabled Spanish version (Supplementary Video S4).

*Surgery:* Offers a curriculum for specific aspects of global health surgery, covering various surgical techniques.

*Intensive care unit:* Presents a comprehensive guide for intensive care training, detailing best practices for respiratory management, intravenous procedures, and patient transition from the operating room to the intensive care unit (ICU).

*Speech therapy:* Introduces the role of Speech-Language Pathology in patient care, covering preoperative and postoperative dysphagia screening.

*Anesthesia:* Focuses on the preparation and execution of bronchoscopy cases, stressing equipment familiarity and best practices.

*Patient or caregiver:* Provides a tailored educational approach for caregivers and patients, covering the entire journey of surgery and postoperative care, aiming to prepare them emotionally and intellectually for the medical process and for parental care responsibilities after discharge.

## 4 Discussion

Global healthcare presents universal challenges that go beyond language and cultural boundaries. This study aims to address these challenges by creating a comprehensive curriculum tailored for a true global collaborative where education can be shared across various languages. The curriculum is built on a decade of research into effective healthcare practices. With a particular focus on Latin America, it emphasizes the importance of a holistic, patient-centered approach to global healthcare. It covers various topics, from surgery and intensive care to guidance for parents and caregivers. This broad scope ensures that the educational material has the potential to significantly impact healthcare professionals and caregivers by providing them with tailored information.

A key innovation of this study is the use of artificial intelligence, specifically generative language models, to surmount existing language and cultural barriers. While our initial focus is on Spanish translations, integrating GLMs allows an easy transition to other languages and cultures. We plan to extend our curriculum to include French, Portuguese, and Arabic translations, aiming for an even greater linguistic and cultural footprint. Additionally, our animated content is designed to mirror global diversity, creating a universal connection that is relatable for learners worldwide. However, incorporating AI into the curriculum development requires oversight due to the potential for inaccuracies and the lack of validation mechanisms (ground truth). In this study, we use native speakers to maintain linguistic precision and cultural nuances.

To evaluate the potential of translations produced by these GLMs, we randomly selected three surgical videos translated by AI and had their original English versions reviewed by medical translators. The translations from the medical professionals and the AI were then assessed by a Spanish-speaking surgeon in Colombia. She reported that both sets of translated videos would provide equal value in supporting her in treating her patients. The Spanish translations completed by the medical professionals were subsequently re-translated into English using ChatGPT and evaluated by a surgeon in Boston. He determined that both the re-translated videos and the original English source material, which had undergone translation and back-translation, were equally informative and offered the same level of support for treating his patients.

There are several potential avenues for enhancing the current workflow. Firstly, the utilization of GLMs for translation can be refined by parallelizing the process and incorporating efficient speech-to-text tools, such as open-source solutions like SpeechBrain, DeepSpeech, or APIs like AWS Transcribe, known for its expertise with medical content. Secondly, the text-to-speech translation can be accelerated by leveraging solutions like ElevenLabs or IBM Watson TTS API.

In conclusion, generative language models can potentially transform global healthcare education, provided they are applied with the necessary safeguards, including data privacy measures and ethical use (31). Our initiative leverages machine learning to create educational resources that are both globally relevant and locally sensitive.

## Data availability statement

The original contributions presented in the study are included in the article/Supplementary material, further inquiries can be directed to the corresponding author.

## Author contributions

FB: Formal analysis, Methodology, Visualization, Writing—original draft. EH: Formal analysis, Methodology, Visualization, Writing—review & editing. EZ: Formal analysis, Project administration, Validation, Visualization, Writing—review & editing. CHe: Formal analysis, Resources, Visualization, Writing—review & editing. KC: Methodology, Validation, Visualization, Writing—review & editing. PV: Formal analysis, Methodology, Visualization, Writing—review & editing. PY: Conceptualization, Formal analysis, Methodology, Supervision, Visualization, Writing—review & editing. CHa: Conceptualization, Formal analysis, Methodology, Supervision, Validation, Visualization, Writing—review & editing.

## Supplementary material

Supplementary Figure S1 Curriculum breakdown.

Supplementary Video S1 Overall Introduction to the Video Curriculum.

Supplementary Video S2 Introducción al video currículum.

Supplementary Video S3 Introduction Nursing Care Home to Hospital to Home.

Supplementary Video S4 Visión general de enfermería UCIP a la sala a casa.
