# Supplementary figures and images for: Overcoming language barriers in pediatric care: a multilingual, AI-driven curriculum for global healthcare education
Source: Front Public Health. 2024 Feb 22;12:1337395. doi: 10.3389/fpubh.2024.1337395 (PMC10917955; doi:10.3389/fpubh.2024.1337395)

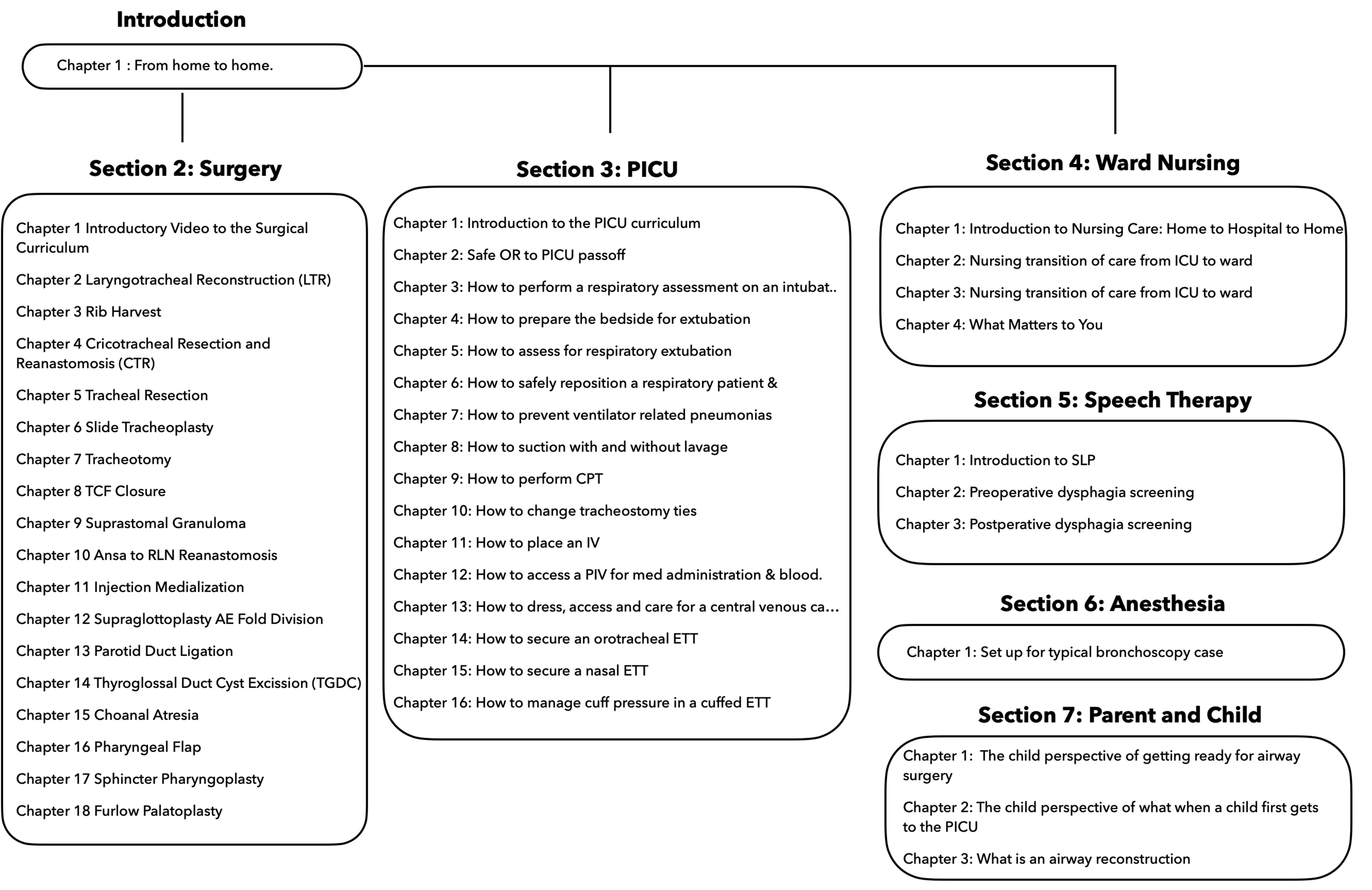

Supplement: Supplementary Figure S1 — Curriculum breakdown. [file Image_1.TIFF]
